# A Dream Fulfilled

**Published:** 1888-06

**Authors:** 


### A DREAM FULFILLED.

A. F. McNeal, a well-known citizen of Rawson, died yesterday, after a short illness, and now comes a weird story connected with this fact, which is as fully authenticated as his death. On the night of the 28th of January of this year, he, like Joseph, "dreamed a dream" that he died and went to heaven. In this dream the date of his death, April 26, was firmly fixed upon his mind. In "the beautiful city, whose maker and builder is God," he dreamed that he met Mr. Mahlon Povenmire, of Ada, an old acquaintance and friend, and asked him when he had died and left earth. Proveinnire replied that he had died and come to the eternal world a week before. There were other striking circumstances in the dream equally as strange, and the next morning when McNeal awoke he reduced the details to writing.

He was then in excellent health. His wife found the manuscript a few days after it was written and it worried the good woman a great deal, but she said nothing to her husband concerning it. Yesterday April 26, McNeal died, while Poveinnire joined the silent majority one week ago yesterday.

The case is a remarkable one, but it cannot be laughed down, for every word of it is true, and the substance of McNeal's dream is in manuscript, just as he wrote it three months ago. It is all the talk in Hancock county.

